# Oral temperature of preweaning dairy heifers: Sensitivity and specificity at detecting individuals with pyrexia

**DOI:** 10.3168/jdsc.2025-0787

**Published:** 2025-07-16

**Authors:** K.R.K. Gottwald, J.A.A. McArt, T. Bhattacharjee, T.E. von Konigslow

**Affiliations:** 1 Department of Population Medicine and Diagnostic Science, Cornell University College of Veterinary Medicine, Ithaca, NY 14853; 2 Department of Computer Science, Cornell University, Ithaca, NY 14853

## Abstract

Change in body temperature can be an early indicator of illness. In calves, body temperature is routinely estimated from rectal temperature measurement. Integrating oral temperature sensors into automated feeding systems might present a feasible alternative to rectal measurement for monitoring calf body temperature. Our objectives were to determine if oral temperature is useful in detecting a fever (rectal temperature ≥39.5°C) and to establish informative oral temperature ranges in dairy calves between 0 and 28 d of life. This diagnostic accuracy study was conducted at one commercial dairy farm in central New York where calves were housed in indoor group pens of 20 calves each with free access to an automated milk feeder. Female Holstein calves (n = 150) were enrolled at birth and followed through 28 d of life. Digital probe thermometers (GLA M700) were used to measure oral and rectal temperatures (10.2 and 5.1 cm probes, respectively) simultaneously at 1, 2, 4, 6, 8, 10, 12, 14, 16, 18, 20, 22, 24, 26 and 28 d of life. Overall mean ± SD (n = 2,081) oral temperature was 38.4°C ± 0.7 and rectal temperature was 38.8°C ± 0.6°C. There was a moderate-to-strong positive correlation (r = 0.75) between oral and rectal temperature. A receiver operating characteristic curve used to evaluate oral temperature in detecting a true fever yielded an area under the curve of 0.91, with a sensitivity of 79% and a specificity of 98% at an oral temperature threshold of ≥39.1°C, as selected by the maximum efficiency index. Our results demonstrate the capability of oral temperature to detect a fever, and that it may be useful for monitoring body temperature changes in preweaning dairy calves. A limitation of our study was that it was conducted in winter and spring with environmental temperatures ranging from −6.7°C to 17.2°C, which mainly fall below calf thermoneutral temperatures (15°C to 25°C). Future work will evaluate the associations of oral temperature with disease and inflammation.

## Highlights

• Oral temperature was evaluated for estimating calf body temperature and detecting fever.
• Oral temperature showed high diagnostic accuracy with reference to rectal temperature.
• Results suggested that oral temperature is an acceptable tool for detecting fever in dairy calves.

## Graphical Abstract

**Figure:**
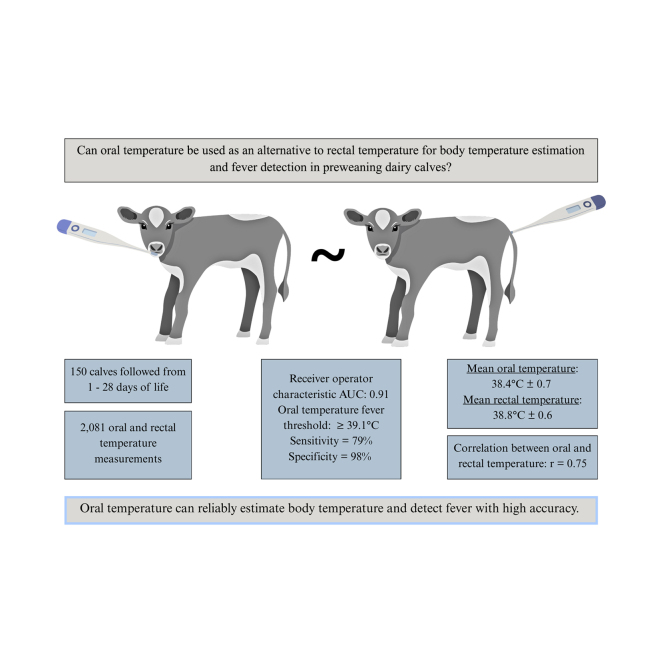
**Summary:** Current automated technologies for measuring calf body temperature are limited in their accuracy and practicality in commercial settings. We hypothesized that oral temperature might be a suitable method for body temperature estimation for automated health monitoring. The objective of this study was to validate oral temperature as an alternative to rectal temperature in preweaning dairy calves, particularly for detecting a fever. One hundred fifty calves were followed from 1 to 28 days of life, having oral and rectal temperatures taken every other day. Oral temperature was typically slightly lower than rectal temperature but reliably reflected systemic changes in body temperature and showed very good diagnostic accuracy at fever detection with a sensitivity of 79% and a specificity of 98%. AUC = area under the curve. Graphical abstract created in Canva by the authors. Calf graphic ©elena-istomina via Canva.com, and thermometer graphic ©sketchify, both via Canva.com.

The preweaning period is a time of high disease risk for dairy calves. Early detection and timely intervention are crucial for preventing disease, reducing its severity, controlling its spread, and enhancing animal welfare. Calf health assessments typically involve the identification of clinical signs and possibly measurement of rectal temperature, which are labor-intensive and may not identify animals showing subtle signs of early illness (Woodrum Setser et al., 2020; Sun et al., 2021). Given the growth and welfare benefits associated with pair and group housing, it is expected that the percentage of preweaning calves raised as part of a group or large population will continue to grow. This shift toward large-group housing emphasizes the need for effective health monitoring systems to direct human attention to animals most in need (Morrison et al., 2021). Advancements in precision technologies and automated health monitoring systems, including accelerometers and automated feeding systems, have improved the ability to track calf health continuously (Costa et al., 2021).

Fever is a key indicator of infection and immune activation (Cimpello et al., 2000; Kirbas et al., 2019) particularly in response to pathogens, which makes it a crucial diagnostic tool (Cimpello et al., 2000). Research regarding fever in neonatal dairy calves often uses the rectal temperature cut-point of ≥39.5°C to define a fever (McGuirk and Peek, 2014; Bell et al., 2020; Costa et al., 2021), which is conserved in this study. Continuous temperature monitoring might allow for earlier disease detection and intervention with the goal of reducing disease severity and treatment costs, and improving calf survival (Vandermeulen et al., 2016). Several technologies to automatically monitor calf body temperature have been explored, including microchips (Woodrum Setser et al., 2020), infrared thermal imaging (Bell et al., 2020; Cantor et al., 2022), rumen temperature boluses (Knauer et al., 2016; Bault, 2023), ear tags, and more (Cantor et al., 2018; Costa et al., 2021). However, there are limitations in their accuracy and practicality of use on farms; all body temperature measurement methods may be influenced by environmental factors; however, estimates from external sites such as the skin, eyes, or ears are especially susceptible to variation due to ambient temperature, wind speed, and sensor placement (Bell et al., 2020). Oral temperature is commonly used in human medicine for body temperature estimation (Smith, 2014; Lei et al., 2021) and may offer a feasible, more accessible, and less invasive alternative to rectal temperature in animal health monitoring. Ideally, oral temperature sensors could be integrated into feeding stations to enable automated measurements of body temperature in preweaning calves to aid in health monitoring while reducing the stress of animal handling and potentially being more “insulated” from ambient conditions than other sites. Since automated milk feeding systems are already being used to monitor feeding behavior as a proxy for health status (Morrison et al., 2021; Sun et al., 2021), we propose that the integration of oral temperature into these systems could be a natural extension to enhance disease detection by providing a more comprehensive profile of calf health.

Our objectives were to (1) determine if oral temperature can detect a rectally diagnosed fever in preweaning dairy calves, and (2) establish baseline oral temperature ranges in preweaning dairy calves between birth and 28 d of life. Our hypothesis was that oral temperature measurement would have a high sensitivity and diagnostic accuracy for fever detection, and that baseline oral temperature ranges would be slightly lower than rectal temperature ranges.

Our diagnostic accuracy study, nested within a prospective observational cohort study, was conducted from February through May of 2024, and STARD reporting guidelines were followed (Cohen et al., 2016). The study protocol was reviewed and approved by the Cornell University Institutional Animal Care and Use Committee (approval no. 2023-0177).

Female neonatal Holstein dairy calves (n = 150) were enrolled from 1 commercial farm in central New York and followed from 1 to 28 d of life. Calf eligibility criteria for enrollment included being female, Holstein, and having survived from the maternity pen to enter the primary calf raising barn. Eligible calves were identified daily from farm birth records and confirmed by their presence in the calf raising barn. Upon eligibility identification, calves were observed for gross head and mouth abnormalities (such as cleft palate, signs of injury from birth, and so on) and were excluded if these were detected. One otherwise eligible calf with a head abnormality was excluded from enrollment. Enrollment occurred in a consecutive series from February to May 2024 until the desired sample size was reached. This sample size was determined using a paired *t*-test power calculation for the concurrent prospective cohort study to detect differences in haptoglobin concentrations between 2 groups, those with and without a fever. The sample size was calculated assuming a mean difference in haptoglobin concentrations between calves with and without fever of 0.1 g/L, an SD of 0.2 g/L (Hajimohammadi et al., 2013), a 1:1 enrollment ratio of calves that do and do not develop a fever, and accounting for a type I error risk of 5% and a type II error risk of 10%. Farm records of calf health were used to estimate how many calves would become clinically ill, and therefore develop a fever. This estimated total enrollment of 134 calves with 67 pyrexic calves anticipated, and we increased this estimate by 10% to account for noncompletion, thus aiming to enroll 150 calves with 75 calves expected to develop a fever.

Calves were born onto a straw-bedded pack, moved to a warming box where they were tube fed 4 L of raw colostrum within 4 h of birth, and then transported to the calf barn within 24 h of life. Calves were housed in small group pens with dimensions of 4 m × 2.5 m that housed 3 to 6 individuals per pen from 1 through 5 d of life, bedded with straw, and offered 2.8 L of warm mixed milk replacer (21% CP, 19% fat; Denkavit, Auburn, NY) via a bottle twice daily. From 5 d of life through the entire observation period, calves were housed in large group pens with dimensions of 9.8 m × 8.9 m that housed 20 individuals per pen, with a straw-bedded pack covering half of the pen and a draining concrete floor for the remainder of the pen. Calves were fed unrestricted pasteurized nonsalable whole milk via an automated feeding station. Calves also had ad libitum access to fresh water and starter pellets (20.91% CP; 5.82% crude fiber, as fed, with Monensin coccidiostat) throughout the entire study. All calves were jacketed while indoor barn temperatures remained below 13°C. Calf mortality and cause of death were documented by farm staff. Calf health assessment and treatment protocols were performed by farm staff using protocols developed with veterinary oversight.

Data collection was planned before the index test (oral temperature) and reference standard (rectal temperature) were measured. Oral and rectal temperature were measured simultaneously at 1, 2, 4, 6, 8, 10, 12, 14, 16, 18, 20, 22, 24, 26, and 28 d of life between 0630 and 1130 h each morning. The frequency of oral and rectal temperature measurements was based upon resource availability. Digital probe thermometers (GLA M700, Agri-Pro Enterprises of Iowa Inc.) were used to measure oral temperature (10.2 cm probe) and rectal temperature (5.1 cm probe) in peak-hold temperature mode until stabilized, which captures the highest temperature recorded during the measurement event. Rectal thermometer probe lengths were selected to reflect those of commonly used rectal thermometers in calves; the oral thermometer probe length was chosen to approximate the length of a standard calf feeding nipple. Calves were quietly approached and manually restrained by cradling their head or hips to minimize stress for oral and rectal temperature measurements. To measure oral temperature, the thermometer probe was inserted straight into the calves' mouths between the tongue and the groove of the hard palate on the medial line until peak temperature was reached (approximately 15 to 20 s). When measuring rectal temperature, the thermometer probe was inserted rectally and held flush against the rectal wall until peak temperature was reached (approximately 15 to 20 s), and measurement was avoided while calves were passing feces or gas to prevent skewed measurements. Observers of index test results were not blinded to reference standard results and vice versa, and all observers were privy to all calves' clinical information. Ambient temperature and fan speed were recorded daily to account for environmental conditions (TC-3, ITC, Seneca Dairy Systems).

Statistical analyses were performed using R Studio (R Core Team, 2023) and Excel (Microsoft Corporation, 2024). All 150 enrolled calves were included in the analyses, but 17 calves died before completion of the 28-d observation period. Death losses occurred between d 5 and 23 of life. Further, 26 total observations with missing data points for index test, reference standard, or both were excluded from the analyses, and all remaining observations were included in the analyses without excluding outliers, yielding 2,081 total paired temperature data points. Descriptive statistics were generated using the *summary* function from the *base* R package. A histogram was created to visualize the distributions of oral and rectal temperatures in relation to each other. Linear regression models were built to assess the correlation between oral and rectal temperatures and ambient temperatures. Pearson correlation coefficient and coefficient of determination were used to quantify the correlation. Receiver operating characteristic (**ROC**) curves were built using *pROC*, version 1.18.5 (Robin et al., 2011) to assess diagnostic accuracy of oral temperature at detecting body temperature conditions based on known values of rectal temperature as the reference standard (hypothermic: ≤38.4°C [Kozat, 2018], normal: 38.5°C to 39.4°C, or hyperthermic: ≥39.5°C [McGuirk and Peek, 2014]). The *OptimalCutpoints* package, version 1.1–5 (López-Ratón et al., 2014), was used to identify useful thresholds of oral temperature. Specifically, the maximum efficiency index was used to select the cut-points, which maximizes the overall proportion of correctly classified cases, accounting for disease prevalence (López-Ratón et al., 2014). A Bland–Altman limits of agreement (**LOA**) plot was generated using the *ggplot2* package (Wickham, 2016) to evaluate agreement between oral and rectal temperature measurements and to assess potential systematic bias.

Overall mean (n = 2,081) oral temperature was 38.4°C ± 0.7°C and mean rectal temperature was 38.8°C ± 0.6°C; 10% (n = 234) of observations were hyperthermic (rectal temperature ≥39.5°C), and 29% (n = 467) of observations were hypothermic (rectal temperature ≤38.4°C). As seen in Figure 1, both oral and rectal temperature values were normally distributed, with oral temperature centered slightly to the left of rectal temperature. Calves with fever had a mean oral temperature of 39.4°C ± 0.6°C and a mean rectal temperature of 39.9°C ± 0.4°C. All observations were included in the analyses, as our goal was to report findings representative of the entire calf population on the dairy farm, including individuals with body temperatures outside biologically typical ranges. No adverse events were observed from performing the index test or reference standard. Mean ambient temperature was 5.9°C ± 5.1°C; minimum and maximum ambient temperatures were 6.7°C and 17.2°C, respectively.

**Figure 1:**
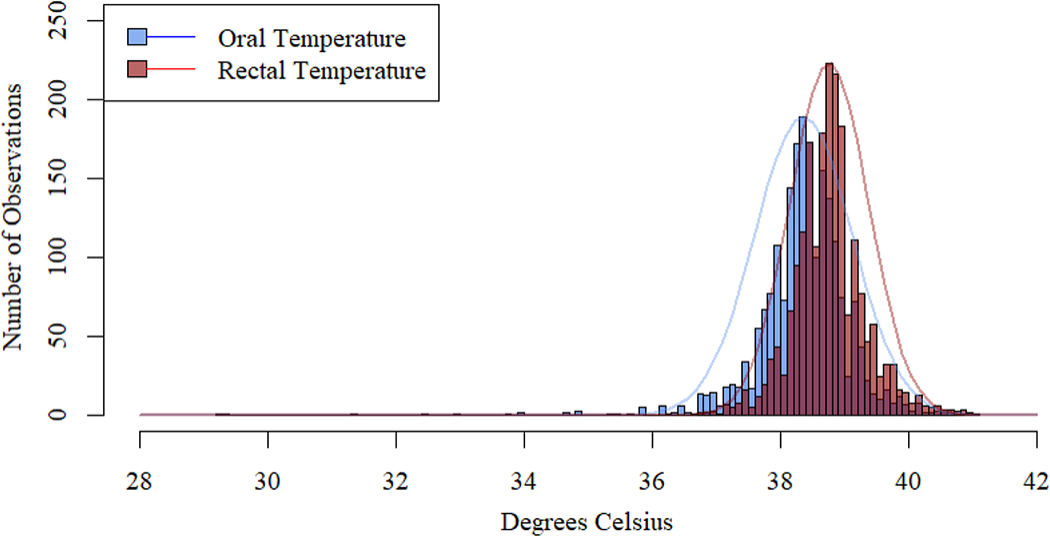
Count and distributions of oral and rectal temperature measurements (°C; n = 2,081) from 150 neonatal replacement heifers (aged 1–28 d) from one commercial dairy herd in New York. Oral temperature measurements are represented in blue, rectal temperature in red.

The linear regression model assessing oral temperature as a function of rectal temperature and ambient temperature showed that oral temperature is a good estimate of rectal temperature (r = 0.92; *P* < 0.01), with each 1°C increase in rectal temperature associated with a 0.92°C increase in oral temperature. Thus, there was a moderate-to-strong positive correlation (r = 0.75, R^2^ = 0.56) between oral and rectal temperature. Ambient temperature had a biologically minor but statistically significant effect on both oral temperature (r = 0.02; *P* < 0.01) and rectal temperature (r = 0.01; *P* < 0.01) such that each 1°C increase in ambient temperature resulted in a 0.02°C increase in oral temperature and a 0.01°C increase in rectal temperature.

An LOA plot was generated to evaluate agreement between oral and rectal temperature measurements (Figure 2). The mean difference between oral and rectal temperatures was −0.4°C, with upper and lower LOA calculated at 0.6°C and −1.4°C, respectively. Approximately 50 observations (3.3% of the dataset) were found to lie outside of the LOA limits, with most of these points located below the lower bound, indicating instances where oral temperature was substantially lower than rectal temperature.

**Figure 2:**
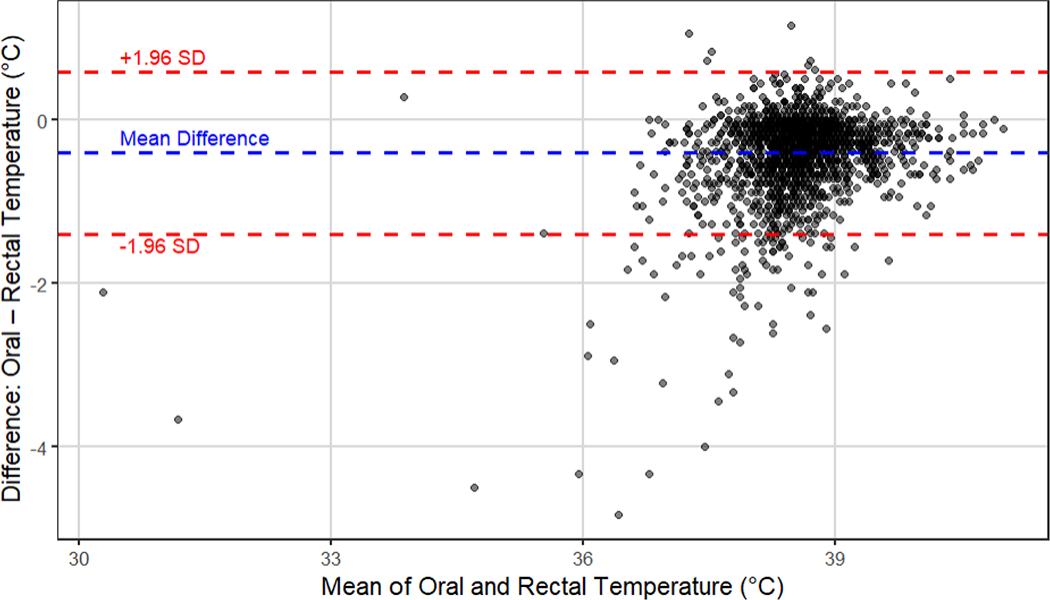
Bland–Altman limits of agreement (LOA) plot showing agreement between oral and rectal temperatures (n = 2,081; mean difference [blue] and 95% LOA [red]) from 150 replacement heifers (aged 1–28 d).

Outcomes from ROC curve analysis of identification of a fever (rectal temperature ≥39.5°C) using oral temperature yielded an area under the curve (**AUC**) of 0.91 (95% CI: 0.88, 0.95), with a sensitivity of 79%, a specificity of 98%, negative predictive value of 0.97, and positive predictive value of 0.80 at an oral temperature threshold of ≥39.1°C. Similarly, an ROC curve of identification of hypothermia (rectal temperature ≤38.4°C) using oral temperature yielded an AUC of 0.85 (95% CI: 0.83, 0.87), with a sensitivity of 75%, specificity of 77%, negative predictive value of 0.92, and positive predictive value of 0.49 at an oral temperature threshold of ≤38.2°C. Selection of each oral temperature cut-point was based on the maximum efficiency index.

The goals of our study were to evaluate the diagnostic accuracy of oral temperature as a tool to detect a fever based on the reference standard of rectal temperature and to describe useful baseline oral temperature ranges in preweaning dairy calves up to 28 d of life. Our results showed a moderate-to-strong positive correlation between oral and rectal temperature across the full spectrum of body temperatures. The R^2^ value suggests that 56% of the variation in oral temperature is explainable by rectal temperature. The partial R^2^ value for ambient temperature suggests that roughly 21% of the remaining variation could be attributed to ambient temperature. Other factors that may contribute to the remaining variation in oral temperature include the observation that the correlation between rectal and oral temperature is stronger when both measurements are within normal body temperature ranges of 38.5°C to 39.4°C (Kozat, 2018) than at extreme values, particularly during severe hypothermia, and the influence of recent consumption of cold water or warm milk on body tissue temperatures (Cantor et al., 2018). Although oral temperature systematically measures slightly lower values than rectal temperature, it demonstrates consistency in detecting fever, with pyrexic calves showing elevated mean oral and rectal temperature values of 39.4°C (±0.6) and 39.9°C (±0.4), respectively. Although measured calf oral temperature is typically lower than measured calf rectal temperature, it reliably reflects systemic changes in body temperature.

The results of the linear regression analysis confirm that rectal and oral temperatures are strongly correlated, and that ambient temperature exerts a statistically significant yet biologically minimal effect. Observations from the research team supported this finding; however, we noted that as ambient temperature dropped further from a calf's thermoneutral zone (15°C to 25°C; Silva and Bittar, 2019), the difference between oral and rectal temperature became greater. Conversely, when close to thermoneutrality, oral temperature aligned more closely with rectal temperature, although it remained slightly lower. The LOA plot showed strong overall agreement between body sites but identified a small subset of measurements (∼3%) outside the limits of agreement. When body temperature was within normal ranges or hyperthermic, oral and rectal readings aligned well; however, when body temperature estimates were below normal, measurements did not agree as well. Datapoints showing oral temperatures far below the 1.96 SD lower limit likely reflect a combination of biological and environmental factors, such as recent cold water intake or severe illness affecting thermoregulation. These findings emphasize the need to interpret oral temperature with caution and use it to guide health monitoring—not as a standalone diagnostic. More research is needed to define context-aware clinical thresholds and explore the value of daily or rolling averages. The influences of ambient temperature, water intake, and other external effects on oral temperature should be further explored, as well as oral temperature dynamics during times of heat stress and across animal management and housing strategies.

The ROC curve analysis demonstrated that oral temperature has excellent diagnostic accuracy in identifying hyperthermia at a cut-point at or above 39.1°C with an AUC of 0.91, showing that oral temperature accurately detects rectally defined fever 91% of the time. Notably, oral temperature has a high specificity for detecting hyperthermia. This may be attributed to the distribution of oral temperature measurements, which typically fall slightly below rectal temperature and rarely exceed it, with exceptions to this likely due to measurement error. This consistent negative difference reduces false-positive fever readings, ensuring that when oral temperature detects a fever, it reliably coincides with a true fever confirmed by rectal temperature, reinforcing its potential as a noninvasive, practical alternative for fever detection.

Similarly, oral temperature is also effective in detecting hypothermia at a cut-point of at or below 38.2°C and an AUC of 0.85, meaning it accurately identifies hypothermia 85% of the time. The temperature stability of oral temperature, which mostly remains within 1°C lower than rectal temperature regardless of ambient conditions, supports its reliability in identifying hypothermic conditions. This stability ensures that oral temperature readings are consistently reflective of true body temperature states. In addition to fever detection, detecting hypothermia is also a useful tool for health monitoring, specifically for identifying calves sick with diarrhea. Unlike the fever typically associated with respiratory disease and other infections, calves with severe diarrhea often exhibit lower body temperatures due to dehydration, energy depletion, and poorer peripheral perfusion to more distal anatomy, which impair the calf's ability to maintain homeostasis (Heller and Chigerwe, 2018). Early detection of hypothermia through oral temperature measurements could enable timely intervention, such as rehydration therapy, supplemental feeding, or warming to prevent further health decline. Overall, oral temperature's consistent performance in detecting both hyperthermia and hypothermia underscores its value in monitoring temperature-related health conditions.

Our study has limitations that should be acknowledged. First, our observational study was performed on one commercial dairy farm, and standard farm disease detection and treatment protocols were maintained throughout the study. Consequently, some sick calves received fluid therapy and anti-inflammatory treatment during the observation period. Although this is beneficial for external validity, the effects of these treatments on oral temperature have not been explored, and it is possible that these treatments influenced oral and rectal temperature differently and thus our results. Although it is possible that the aforementioned treatments do not influence oral and rectal temperature equally, the authors hypothesize that, because body temperature is regulated systemically, the impact of these treatments on oral and rectal temperature is likely to be very similar. Next, our statistical analysis was designed to assess the diagnostic accuracy of oral temperature measurements. Future analyses will account for repeated measures when looking at associations with health. Last, most observations took place in conditions below calves' thermoneutral zone of 15°C to 25°C (Silva and Bittar, 2019). As discussed previously, ambient temperature does have an influence on oral and rectal temperature, and on the difference between the 2 measurements. Therefore, exploring oral temperature in conditions above and within calves' thermoneutral zone would be an important next step. Future studies should investigate the relationships of oral temperature with variable environmental conditions, disease and inflammatory states, and housing conditions. Also, the influence of ambient conditions on body temperature may have led to classification errors; ambient temperature may have affected classification of fever status, particularly in borderline cases, and could have contributed to misclassification of calves' fever status in our analyses. This should be considered when interpreting the results and in future studies using fixed temperature thresholds for disease detection. Continued work should also include prototype sensor development and integration into automated milk feeders and health monitoring systems for frequent measurements of oral temperature throughout the day to investigate diurnal variability.

Our results establish that the index test, oral temperature, is an acceptable tool for detecting fever as defined by the reference standard of rectal temperature, and that oral temperature has potential to be a useful metric in automated health monitoring of preweaning dairy calves. Oral temperature showed excellent diagnostic accuracy at detecting a fever and very good diagnostic accuracy at detecting hypothermia. Oral temperature consistently detected abnormal body temperature status in neonatal dairy calves. Although rectal temperature will likely remain the gold standard for clinical assessments, oral temperature offers a practical and accessible alternative to rectal temperature, making it a valuable tool to enhance animal health monitoring. By providing an additional means to identify calves in need of more thorough physical examination and intervention, oral temperature information can help guide animal caretakers in prioritizing calves that require further attention. Our goal is not to replace rectal temperature measurements as part of a physical examination, but rather to integrate oral temperature into automated health monitoring systems and to supplement other health data with a reliable, accessible measurement of body temperature, which can help improve overall animal health management and facilitate early intervention strategies.
